# Cilia and transcription: a mini review

**DOI:** 10.3389/fcell.2025.1582796

**Published:** 2025-06-09

**Authors:** Jason M. Brown

**Affiliations:** Department of Biology, Salem State University, Salem, MA, United States

**Keywords:** cilia, flagella, transcription, *Chlamydomonas*, FoxJ1, RFX, XAP5, gene regulation

## Abstract

Cilia assembly is accompanied by rapid and highly coordinated transcription of hundreds of genes. Cilia gene regulation has been studied extensively in both metazoans and unicellular model organisms. The forkhead and RFX family transcription factors regulating cilia genes in animals were first identified 25 years ago and considerable molecular details of the regulatory processes have been described since then. While many of the most important early studies of cilia gene regulation were done in unicellular organisms, additional molecular players need to be discovered for a more complete understanding in these organisms. In this concise review, written primarily for students new to the field, I present a brief history of research on cilia gene regulation, highlight some key metazoan discoveries from the last decade, and discuss gaps in our understanding of cilia gene regulation in unicellular model organisms with a focus on *Chlamydomonas reinhardtii*.

## 1 Introduction

Cilia (a.k.a. Flagella in some contexts) extend from eukaryotic cells to carry out important functions in motility and sensory perception. Cilia are complex organelles including a ciliary membrane with distinct composition surrounding a ciliary matrix and a microtubule-based axoneme (Brown and Witman, 2014; Mill et al., 2023). The requirement for nodal cilia in determining left-right asymmetry during vertebrate development (Nonaka et al., 1998) and for primary cilia in signaling pathways critical for development and homeostasis (Pazour et al., 2000) led to intense interest in cilia over the last quarter century (Brown and Witman, 2014; Mill et al., 2023). Research on cilia includes studies on the regulated expression of hundreds of genes encoding cilia proteins (hereafter ‘cilia genes’). This review focuses on the historical and recent discoveries in cilia gene regulation with emphasis on metazoan transcription factors and underexplored regulatory mechanisms in unicellular organisms, particularly *Chlamydomonas reinhardtii*. As the mini-review format makes it difficult to cite all relevant literature, I apologize to authors I have excluded and direct interested readers to other excellent reviews describing cilia gene expression in more depth (Choksi et al., 2014; Lefebvre and Rosenbaum, 1986; Lewis and Stracker, 2021; Thomas et al., 2010).

## 2 A brief history of research on cilia and transcription

Research on expression of cilia genes can be viewed as a series of convergences. Discoveries on gene expression occurred in parallel with new cilia discoveries until these paths converged and led to critical breakthroughs in cilia gene expression.

### 2.1 Connecting cilia and gene expression across phyla

The 1960s were an important period for research on cilia and transcription. The foundation for understanding gene expression was established when theoretical and experimental approaches to DNA structure and function (Crick, 1958; Dounce, 1953; Franklin and Gosling, 1953; Watson and Crick, 1953) and genetic approaches to gene regulation and the genetic code (Crick et al., 1961; Jacob and Monod, 1961) converged at the beginning of the decade with the discovery of messenger RNA (Brenner et al., 1961; Cobb, 2015; Gros et al., 1961; Matthaei and Nirenberg, 1961). Microscopy and biochemistry of cilia identified axonemal dynein arms as the location of the ATPase for ciliary motility (Gibbons, 1963; Gibbons and Rowe, 1965). Early studies on cilia growth were in *Chlamydomonas moewusii* and sea urchin (Auclair and Siegel, 1966; Lewin, 1953). Soon after gene expression principles were discovered, studies on ciliary regeneration demonstrated its value as a model system for regulated gene expression (Rosenbaum and Child, 1967). Importantly, cilia regeneration and incorporation of tritiated leucine into TCA insoluble protein were inhibited by cycloheximide, making it clear that new protein synthesis is required for assembly of full-length cilia (Rosenbaum and Child, 1967).

Bookending the decade, Rosenbaum et al. (1969) published a watershed paper establishing *C. reinhardtii* as a model organism for cilia gene regulation. When cycloheximide inhibited protein synthesis during deciliation, cells formed half-length cilia, indicating a preexisting pool of cilia proteins. This also suggested that a reduced concentration of one or more proteins in this pool might be a limiting factor for full-length cilia assembly (Rosenbaum et al., 1969). Pulse labeling of arginine-requiring cells with arginine-^3^H during cilia regeneration showed that incorporation of newly synthesized proteins into cilia peaked when the precursor pool in the cell body was nearly depleted. Colchicine inhibition of cilia growth also indicated that new protein synthesis could occur in the absence of cilia elongation (Rosenbaum et al., 1969). Later work showed that this increase in cilia protein synthesis is largely due to mRNA accumulation (Minami et al., 1981; Silflow and Rosenbaum, 1981). Additional progress in *Chlamydomonas* is discussed in more detail below, especially in Section 4.

Early studies on cilia regeneration in *Tetrahymena* and sea urchin showed that ability to regenerate cilia is conserved across phyla (Auclair and Siegel, 1966; Rosenbaum and Carlson, 1969). Unlike in *Chlamydomonas*, cycloheximide treatment of *Tetrahymena* at the time of deciliation completely blocked cilia regeneration suggesting that synthesis of some limiting protein is necessary to utilize the cilia precursor pool present at the time of deciliation (Guttman and Gorovsky, 1979; Rannestad, 1974; Rosenbaum and Carlson, 1969). Cilia protein synthesis in starved regenerating *Tetrahymena* cells can be detected above the protein synthesis background in starved non-deciliated cells revealing a dramatic increase in tubulin synthesis (Guttman and Gorovsky, 1979). This increase in tubulin synthesis is preceded by an increase in tubulin gene transcription resulting in increased tubulin mRNA available for translation (Bird and Zimmerman, 1980; Guttman and Gorovsky, 1979). Cilia regeneration in sea urchin embryo does occur in the presence of the protein synthesis inhibitor puromycin, indicating that, like in *Chlamydomonas*, a preexisting pool of cilia precursor proteins can be used in the absence of translation (Auclair and Siegel, 1966). However, new protein synthesis, including synthesis of tubulin and other less abundant cilia proteins, is induced during regeneration (Stephens, 1977). Increased tubulin mRNA abundance could be blocked by the transcription inhibitor actinomycin D (Merlino et al., 1978), and nuclear run-on assays confirmed that increased tubulin mRNA abundance during cilia regeneration is due in part to increased transcription (Gong and Brandhorst, 1987).

The dramatic transformation of *Naegleria gruberi* from amoeba to an elongated cell with cilia that occurs on transferring cells to nutrient-free media (Fulton and Dingle, 1967) was also developed during this period as a model to study cilia gene expression. The majority of tubulin incorporated into the growing cilia is newly synthesized during this differentiation period (Kowit and Fulton, 1974) and is specific to cilia (Kennard et al., 2025). Differentiation-specific tubulin synthesis can be inhibited by actinomycin D (Fulton and Kowit, 1975) and translatable tubulin mRNA increases during differentiation (Lai et al., 1979). Nuclear run-on assays showed that the increase can largely be attributed to increased transcription (Lee and Walsh, 1988). Application of microarrays and RNA-seq to study differentiation-specific gene induction in *Naegleria* revealed increased expression of multiple cilia genes including a cilia-specific network of microtubule binding proteins (Fritz-Laylin and Cande, 2010; Kennard et al., 2025). Taken together, the studies described above established non-vertebrate model organisms as important contributors to a broad understanding of cilia gene regulation.

### 2.2 Connecting cilia to specific transcription factors

Work in the late 1990s implicated specific transcription factors in the regulation of cilia genes. Interest in cilia expanded after mouse embryonic nodal cilia were shown to be required to establish normal left-right asymmetry (Nonaka et al., 1998) and cilia were first connected to polycystic kidney disease (Pazour et al., 2000; Qin et al., 2001). Initial characterization of transcription factors in the 1980s led to studies of tissue specific expression patterns in the 1990s (Clevidence et al., 1993; Maniatis et al., 1987). The first transcription factor shown to co-occur with cilia assembly in both space and time was mammalian HFH-4 (FOXJ1) (Blatt et al., 1999; Hackett et al., 1995) a forkhead family protein which is also required for mouse motile cilia assembly (Chen et al., 1998). Its orthologs in *Drosophila* and *Caenorhabditis elegans* regulate specialization of cilia in sensory neurons (Cachero et al., 2011; Newton et al., 2012, Brocal-Ruiz et al., 2023).

The RFX protein, DAF-19, was identified in a screen for mutants with defective dye filling in sensory cilia in *C. elegans* (Perkins et al., 1986) and was found to regulate cilia genes *via* a 14-bp target sequence, the X box (Swoboda et al., 2000). RFX genes have only been found in the genomes of unikont organisms. However, not all ciliated unikonts have RFX transcription factors and some non-ciliated unikonts do have RFX. Piasecki et al. (2010) concluded from these observations that RFX originated in an early unikont ancestor and only later began regulating cilia genes in an early animal or ancestor of animals and choanoflagellates. The important recent discovery that choanoflagellates also use RFX to regulate cilia genes clearly pushes this event back to a common ancestor of choanoflagellates and animals (Coyle et al., 2023). In addition, some lineages secondarily lost cilia and maintained RFX which regulates non-cilia genes. Additional details on FOXJ1 and RFX regulation are discussed below.

### 2.3 Cataloging cilia genes

Understanding cilia gene regulation needs to include identifying which genes are regulated. Early studies developing methods for purifying and fractionating cilia in *Tetrahymena* and *Chlamydomonas* began to reveal the complexity of the cilia proteome (Gibbons, 1963; Piperno et al., 1977; Witman et al., 1972). Genome sequencing allowed comparison of genomes of ciliated and non-ciliated organisms identifying hundreds of candidate cilia genes (Avidor-Reiss et al., 2004; Li et al., 2004; Merchant et al., 2007). A recent study using additional tools now available for phylogenetic profiling identified 152 new strong candidate cilia genes (Dobbelaere et al., 2023). Genome-wide searches and comparative genomics identified multiple new cilia genes with X box sequences in their promoters in *C. elegans* (Blacque et al., 2005; Chen et al., 2006; Efimenko et al., 2005) and *Drosophila* (Laurençon et al., 2007). Proteomic analyses of purified cilia added more cilia proteins and linked them with specific cilia subfractions (Ishikawa et al., 2012; Pazour et al., 2005; Smith et al., 2005). As a complementary approach, transcriptomics supported many of the results of the earlier studies and added additional cilia genes. Some of these studies attempted to establish a transcriptional profile during normal or experimentally induced ciliogenesis (Albee et al., 2013; Fritz-Laylin and Cande, 2010; Kennard et al., 2025; Patir et al., 2020; Quigley and Kintner, 2017; Stolc et al., 2005; Zones et al., 2015). In others, transcriptome analysis was performed after the expression of RFX or forkhead transcription factors was disrupted or induced ectopically to identify genes regulated by the transcription factors in question (Chung et al., 2014; Lemeille et al., 2020; Phirke et al., 2011; Stubbs et al., 2008). Various studies have also made ciliome databases accessible to the research community including CilDB, Syscilia Gold Standard, CiliaCarta, and CilioGenics, and the *Chlamydomonas* flagellar proteome database (Arnaiz et al., 2014; Elliott et al., 2023; Pazour et al., 2005; Pir et al., 2024; van Dam et al., 2013; van Dam et al., 2019).

## 3 Expanding understanding in metazoans

Involvement of FOXJ1 and RFX family transcription factors in the expression of animal cilia genes was discovered over 20 years ago (Choksi et al., 2014). FOXJ1 regulates the expression of proteins needed for motility including dynein arms and radial spokes (Lewis and Stracker, 2021). RFX family transcription factors are more generally involved in transcription of proteins needed for assembly of both motile and immotile cilia (Choksi et al., 2014). Considerable details have also emerged about functional specialization of cilia through the use of different combinations of transcription factors and their upstream regulatory networks.

### 3.1 RFX and forkhead interactions in functional specialization of cilia

Interactions between FOXJ1 and RFX family transcription factors have become increasingly clear during the development of specific ciliary functions in different contexts. Correct specification of ciliated dendrites on the chordotonal neurons in *Drosophila* is dependent on both RFX and the distant FOXJ1 relative, fd3F (Cachero et al., 2011; Newton et al., 2012). Similarly, *C. elegans* FKH-8 (a forkhead TF) and DAF-19 (an RFX TF) interact with each other in regulation of cilia genes for assembly of sensory cilia (Brocal-Ruiz et al., 2023). Surprisingly, mouse FOXJ1and *Xenopus* FOXN4 are able to functionally substitute for FKH-8 for *C. elegans* cilia gene expression, suggesting that co-regulation of cilia genes by RFX and forkhead proteins may be an ancient connection. Human RFX3 and FOXJ1 act together in human airway MCCs (Didon et al., 2013) as described in more detail below. RFX2 interacts with FOXJ1 for cilia gene expression in larval MCCs in *Xenopus*. In these interactions FOXJ1 may be most often bound to distal enhancers while RFX2 is bound to proximal promoter sequences and recruits FOXJ1 to the promoters by dimerization and chromatin looping (Quigley and Kintner, 2017). It is unknown whether similar interactions occur in vertebrates outside of motile ciliogenesis. A more detailed description of the specific gene regulatory network in MCC differentiation is presented below.

### 3.2 Multiciliogenesis

**FIGURE 1:**
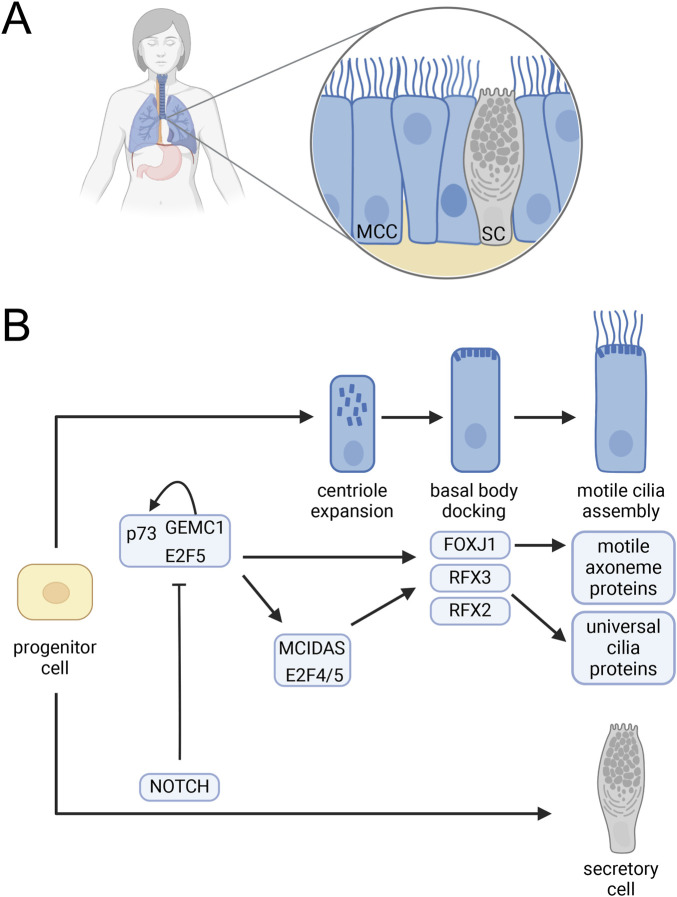
Multiciliated cell differentiation. **(A)** Mammalian respiratory epithelium including MCC interspersed with secretory cells. **(B)** Regulation of MCC development. A complex containing GEMC1 and the E2F5 and p73 transcription factors activates p73 as well as MCIDAS and the FOXJ1, RFX2 and RFX3 ciliogenesis transcription factors. MCIDAS in complex with E2F4 or E2F5 also activates FOXJ1, RFX2, and RFX3 and promotes centriole expansion. FOXJ1 promotes basal body docking and activates expression of axonemal proteins required for cilia motility. RFX2 and RFX3 activate the expression of many cilia genes needed for assembly of all cilia including the motile cilia on MCC. Notch signaling blocks GEMC1 complex activation leading to a secretory cell fate (Lalioti et al., 2019; Lewis and Stracker, 2021; Lu et al., 2019). Created in BioRender. Brown, (2025) https://BioRender.com/z01l282.

In humans and mice, cells with dozens of motile cilia (multiciliated cells, MCC) are found on the respiratory epithelium (Figure 1A), in brain ventricles, and in reproductive organs (Brooks and Wallingford, 2014). During commitment of progenitor cells to MCC differentiation, the expression of FOXJ1 and RFX2/3 is tightly regulated. Three key regulators of FOXJ1 and RFX expression are the geminin family proteins GEMC1 and MCIDAS (a.k.a. multicilin) and the transcription factor p73 (Lu et al., 2019; Marshall et al., 2016; Terré et al., 2016). Despite tissue-specific differences in these regulatory pathways (Wildung et al., 2019), a relatively clear picture has emerged for respiratory and oviduct epithelia (Figure 1B) (Lewis and Stracker, 2021). In these tissues, GEMC1 acts upstream of MCIDAS (Lu et al., 2019) and forms a complex with p73 and the transcription factor E2F5. This complex regulates the expression of downstream regulators of cilia genes including FOXJ1, RFX3, and p73, itself, such that loss of either GEMC1 or p73 led to loss of respiratory and oviduct MCCs (Lalioti et al., 2019). MCIDAS acting downstream of GEMC1 and in a complex with E2F4 or E2F5 activates FOXJ1 and other genes involved in the extensive basal body duplication needed to nucleate multiple cilia (Lu et al., 2019). The story in ciliated cells in the brain is somewhat less clear. However, loss of p73 in the choroid plexus leads to an upregulation of E2F/MCIDAS activity and upregulation of microRNA-449 which compensates for the absence of p73 (Wildung et al., 2019). Additional work will be necessary to fully describe these interactions in brain epithelial cells.

### 3.3 Other emerging regulatory mechanisms

#### 3.3.1 Auto fatty acylation and dimerization

Using recombinant RFX3 in the presence of clickable fatty acid analogs Chen et al. (2018) found that RFX3 autoacylates at a conserved cysteine (544) in its dimerization domain. Mutation of this cysteine reduced fatty acylation and dimerization but not nuclear localization. In an RFX3 null background, expression of WT RFX3, but not C544S RFX3 drove the expression of three known RFX3 targets, suggesting that RFX3 fatty acylation is required for normal expression of cilia genes (Chen et al., 2018).

#### 3.3.2 Regulation by corticosteroid receptors

ChIP-seq analysis in the rat hippocampus identified over 50 cilia genes bound by mineralocorticoid receptors (MRs). MRs normally respond to circadian and stress-induced changes in adrenal gland hormone release (Mifsud et al., 2021). MR bound at sites on the DNA that are also bound by RFX3, suggesting a possible RFX3/MR interaction. Supporting that hypothesis, MR agonists were required for neuronal differentiation and ciliogenesis and MR antagonists prevented differentiation and cilia growth (Mifsud et al., 2021). Together these results suggest that a response to corticosteroids through MR and RFX3 is required for ciliogenesis involved in neuronal development.

#### 3.3.3 Mitochondrial stress

Recent studies have indicated a signaling connection between mitochondria and cilia (Bae et al., 2019). Perturbing the function of mitochondria in astrocytes led to robust activation of multiple cilia genes and FOXJ1 (Ignatenko et al., 2022). The astrocytes remained monociliated despite the role of FOXJ1 in multiciliogenesis in other contexts. However, the cilia were abnormally long and contorted due to an unidentified mechanism. It is possible that the upregulation of cilia genes only occurs as a pathological response (Ignatenko et al., 2022), but it will be important to explore the intriguing possibility that mitochondrial function might be connected to cilia gene regulation under normal circumstances.

## 4 Cilia and transcription in *Chlamydomonas*

**FIGURE 2:**
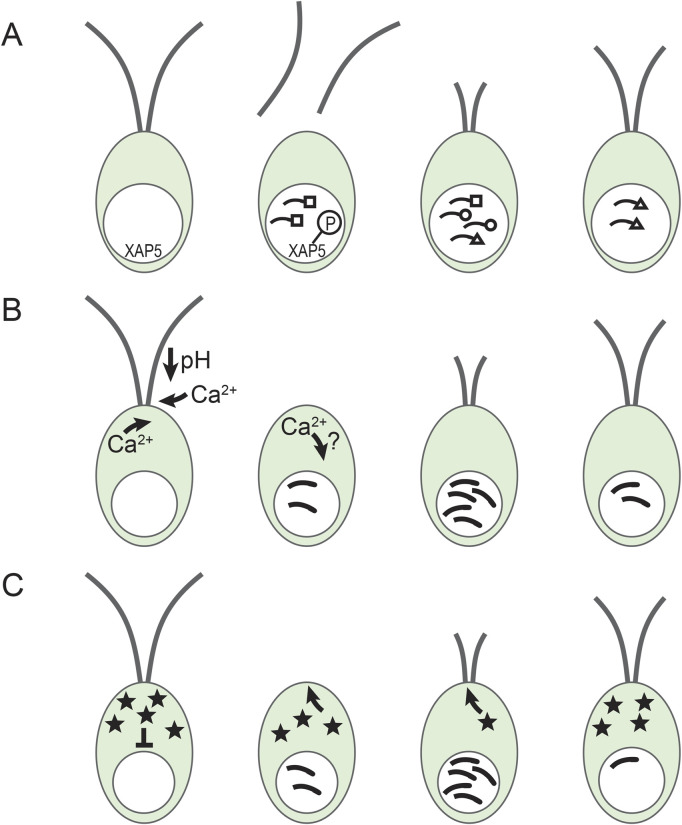
Gene regulation during cilia regeneration in *Chlamydomonas*. **(A)** Different temporal expression patterns and XAP5. Cilia genes are expressed in groups with different expression patterns. Depicted here are early (squares) middle (circles) and late (triangle) genes (Schloss et al., 1984). In addition, nuclear localized XAP5 is phosphorylated rapidly after deciliation by an unidentified kinase. **(B)** Calcium involvement in cilia gene induction. Upon pH shock, extracellular calcium enters the cell and stimulates the release of additional calcium from intracellular stores and triggers deciliation (Quarmby and Mahjoub, 2023). Calcium entry is needed for maximal gene induction concomitant with the initiation of cilia regrowth (Cheshire and Keller, 1991). How the intracellular calcium concentration changes are involved in normal gene induction is still unclear. **(C)** The repressor sequestration model. A constitutively produced repressor (stars) blocks cilia gene expression. After deciliation, the repressor is sequestered in rapidly growing cilia, reducing the effective concentration of the repressor in the cell body and allowing increased transcription of cilia genes. As cilia approach full length, cilia assembly slows down allowing the repressor to accumulate and cilia gene expression to slow down (Perlaza et al., 2023).

During *Chlamydomonas* cilia regeneration, new protein synthesis begins within the first few minutes and peaks around the time that the pre-existing cilia precursor pool in the cell body would be depleted without translation (Lefebvre et al., 1978; Rosenbaum et al., 1969; Weeks et al., 1977). This increase in translation corresponds with a peak in cilia gene mRNA abundance as shown initially for tubulin genes (Minami et al., 1981; Silflow and Rosenbaum, 1981). The mechanisms of the increases and decreases in mRNA abundance remain largely unknown although some of the story has been revealed (Figure 2).

### 4.1 Message abundance changes

#### 4.1.1 Increased transcription and changes in message stability

The increase in mRNA abundance is due to increases in both transcription and mRNA stability. Run-on transcription of tubulin RNAs increased in nuclei isolated from cilia regenerating cells compared with nuclei from non-regenerating cells. This was the first direct demonstration that new transcription contributes to tubulin mRNA accumulation during regeneration (Keller et al., 1984). Baker et al. (1984) confirmed this result with *in vivo* ^32^P pulse labeling and found that tubulin mRNA stability is doubled in deciliated cells compared with non-deciliated cells. Cis DNA sequence elements have been identified that regulate increased expression during cilia regeneration of the *TUB2* β-tubulin gene, the *TUA1* α-tubulin gene, and the *DIC2* (*a.k.a. ODA6*) gene encoding flagellar outer arm dynein intermediate chain 2 (a.k.a. IC70) (Bandziulis and Rosenbaum, 1988; Davies et al., 1992; Davies and Grossman, 1994; Kang and Mitchell, 1998; Periz and Keller, 1997). After cilia regenerate, the stabilization of tubulin mRNAs switches to rapid degradation, a translation-dependent process that is independent of the normal deadenylation-dependent pathway operating on the same mRNAs prior to deciliation (Baker et al., 1986; 1984; Baker and Liggit, 1993; Gera and Baker, 1998). Thus, increases in transcription and mRNA stability both contribute to the accumulation of cilia gene mRNAs and the decrease in message abundance following regeneration involves a novel pathway that has not yet been characterized.

#### 4.1.2 Complex patterns of mRNA abundance change

Although expression of most cilia genes increases minutes after deciliation, timing is different for different groups of genes (Albee et al., 2013; Lefebvre et al., 1978; Remillard and Witman, 1982; Schloss et al., 1984). Lefebvre et al. (1978) found that tubulin synthesis remained high for hours while synthesis of other proteins peaked in under an hour. Remillard and Witman (1982) used 2-D gels to connect proteins to specific ciliary structures (e.g., radial spokes) and showed that proteins found together in a certain structure were produced with similar kinetics. Subsequently, dot blot hybridization identified three classes of RNAs that were increased at early, middle, and late times during regeneration (Figure 2A) (Schloss et al., 1984). Albee et al. (2013) using RNA-seq identified 16 different expression profiles. Again, genes encoding proteins that work together often had a similar expression pattern (Albee et al., 2013). It is likely that these complex expression patterns involve several parallel regulatory pathways that have yet to be discovered.

It is unclear whether the mechanisms regulating cilia gene expression during the cell cycle are the same as those during cilia regeneration. *Chlamydomonas* cells in synchronized cultures resorb their cilia around the G1/S transition and regrow them with most cells being ciliated by the transition to the post-mitotic phase. Levels of mRNAs encoding intraflagellar transport (IFT) proteins were strictly regulated with peak accumulation occurring during cilia assembly in S/M (Wood et al., 2012). RNA-seq analysis during the diurnal cycle showed that many cilia genes are coordinately regulated during cilia assembly and exhibit different expression clusters (Zones et al., 2015). However, since these studies do not have the same temporal resolution as cilia regeneration studies (Albee et al., 2013) it is unknown whether the regeneration expression clusters are relevant to cilia growth during the cell cycle. Interestingly, some mutants induce cilia genes in cycling vegetative cells but not in non-cycling gametes, suggesting distinct cilia gene regulation during the cell cycle (Lefebvre et al., 1988).

### 4.2 Transcription factors

Importantly, the FOXJ1 and RFX transcription factors are not found in *Chlamydomonas* (Chu et al., 2010; Piasecki et al., 2010). Recently, the first transcription factor, XAP5, regulating *Chlamydomonas* cilia genes was identified (Li et al., 2018). Consistent with the hypothesis that multiple pathways control cilia genes, *xap5* mutant cells downregulated expression of some cilia genes whereas others were unaffected. Nuclear localization of XAP5 was needed for expression of XAP5-dependent genes (Figure 2A). Sequence-specific binding of XAP5 to cilia gene promoters assisted in the recruitment of RNA polymerase II to those promoters indicating that XAP5 is a transcription factor (Li et al., 2018). Although XAP5 is required for basal expression of several cilia genes and for upregulation of some of those genes following pH shock, it is currently unclear whether XAP5 is required for the normal induction of cilia genes during regrowth since the *xap5* mutant lacks cilia.

While *XAP5* orthologs are found broadly in eukaryotes, their presence in a genome does not predict presence of cilia and so far, XAP5 has only been shown to regulate cilia genes in *Chlamydomonas* and mice. In mice, XAP5 and its paralog XAP5L act antagonistically during spermatogenesis with XAP5 promoting expression of multiple cilia genes including FOXJ1 and RFX transcription factors, whereas XAP5L represses many of those same genes (Wang et al., 2025). The *Naegleria gruberi* and *Tetrahymena thermophila* genomes both lack *XAP5* (Eisen et al., 2006; Fritz-Laylin et al., 2010; Tegenfeldt et al., 2025). Emphasizing that XAP5 proteins have other functions outside of regulating cilia genes, *Schizosaccharomyces pombe* which lacks cilia, does have an *XAP5* orthologous gene in its genome (Tegenfeldt et al., 2025; Wood et al., 2002).

Since XAP5 only regulates some cilia genes, additional transcription factors likely regulate the XAP5-independent *Chlamydomonas* cilia genes. Three other predicted transcription factors have been suggested as good candidates due to their increased expression following deciliation and during cell cycle regulated cilia growth (Albee et al., 2013; Sale and Dutcher, 2023; Zones et al., 2015). These are Cre02. g103450, encoding a protein with a domain 50%–60% similar over 100 amino acids to MYB domains in plants and animals, Cre03. g201250 encoding a protein with zinc finger and G-patch nucleic acid binding domains, and Cre04. g228400, encoding a WRKY family plant and algae specific transcription factor (Craig et al., 2023; Sale and Dutcher, 2023). Interestingly, although Cre03. g201250 and Cre04. g22840 mRNA levels peaked around the time that most cells are ciliated, Cre02. g103450 level remained elevated over the next 10 h of day in light-synchronized cultures (Zones et al., 2015). This difference suggests possible functional differences between Cre02. g103450 and the other two genes. It will be important to analyze cilia gene expression during regeneration and the cell cycle in mutants lacking these proteins.

### 4.3 Signaling pathways

Upregulation of cilia genes in *Chlamydomonas* is likely to involve initiating events associated with cilia that stimulate signaling to the nucleus activating one or more transcription factors. The initiating signal and molecular details of the signaling pathway(s) are still not known, but some clues have emerged and are discussed below (Quarmby and Mahjoub, 2023; Sale and Dutcher, 2023).

#### 4.3.1 Calcium

Calcium signaling regulates cilia motility, pH shock deciliation, cilia growth and maintenance, and cilia gene expression (Brown et al., 2012; Cheshire and Keller, 1991; Quader et al., 1978; Quarmby and Mahjoub, 2023). Under normal conditions, pH shock or mechanical shearing lead to deciliation, selective accumulation of mRNAs from cilia genes, and regrowth of cilia. Manipulating calcium concentration uncoupled these events (Cheshire and Keller, 1991). Cells deciliated in 10^−7^ M calcium did not regenerate cilia or accumulate cilia mRNAs until calcium was added, after which submaximal accumulation of cilia mRNAs occurred. When calcium was present during deciliation but lowered immediately after, a submaximal cilia mRNA accumulation occurred after deciliation that was uncoupled from cilia growth. If these cells are maintained in ∼10^−7^ M calcium, they begin to regrow cilia around 135 min after deciliation. This regrowth was accompanied by a small peak of mRNA abundance (Cheshire and Keller, 1991). In addition, wild-type cells accumulate cilia mRNAs in response to experimentally stimulated cilia resorption and mutants lacking cilia or unable to deciliate and cells treated to block calcium influx accumulate cilia mRNAs submaximally (Cheshire et al., 1994; Evans and Keller, 1997; Lefebvre, 1980; Lefebvre et al., 1978). Together these results indicate that maximal cilia gene induction is likely to result from a combination of responses from overlapping signaling pathways, some of which are calcium dependent (Figure 2B) (Cheshire and Keller, 1991).

#### 4.3.2 Kinases

Many signaling pathways involve kinases that add phosphate groups to modify the activity of target proteins. Several *Chlamydomonas* cilia associated kinases have been identified (Mahjoub et al., 2004; Pazour et al., 2005; Wang et al., 2019; Wilson and Lefebvre, 2004). However, no kinases have yet been definitively connected with cilia gene induction. Interestingly, nuclear localized XAP5 transcription factor was phosphorylated within 1 min after pH shock deciliation (Figure 2A) and mutant XAP5 that could not be phosphorylated did not support cilia growth (Li et al., 2018). Identifying the XAP5 phosphorylating kinase could be an important step in identifying signaling pathways for cilia gene induction during regeneration.

#### 4.3.3 Repressors

An early response leading to cilia gene induction could be activation of an activator protein or inactivation of a repressor protein. Perlaza et al. (2023) recently proposed and tested two repressor-based models. Their repressor sequestration model postulated that a repressor, or a protein that activates a repressor, is continually produced and is preferentially sequestered in rapidly growing cilia (Figure 2C). This model reproduced the essential features of cilia length control and cilia gene expression dynamics. It also correctly predicted that mutants with impaired IFT transport have lower accumulation of cilia mRNAs. If alleviation of repression is a fundamental mode of upregulation for the pulse of cilia genes, identifying the postulated repressor will be a critical next step.

## 5 Discussion

Sixty years ago, cilia regeneration was established as a model for gene induction (Rosenbaum and Child, 1967). Recently, much has been learned about the transcription factors FOXJ1, RFX2, and RFX3, including upstream signaling pathways, interactions with other transcription factors, and tissue-specific differences in regulation (Lewis and Stracker, 2021). However, many tissue-specific details need clarification. For instance, how microRNAs and p73 coordinate MCC differentiation in the choroid plexus is still not completely understood. Autoacylation of RFX3 also raises the question of what other post-translational modifications are important.

Cilia gene regulation in unicellular organisms is primed for exploration (Marshall, 2024; Quarmby and Mahjoub, 2023; Rosenbaum, 2009; Sale and Dutcher, 2023). FOXJ1 and RFX-family proteins are absent in *Chlamydomonas*, *Tetrahymena*, and *Naegleria*. (Piasecki et al., 2010). *XAP5* orthologs are found broadly in eukaryotes, but not in all ciliated organisms. For instance, the *Naegleria* and *Tetrahymena* genomes lack *XAP5* (Tegenfeldt et al., 2025). Other transcription factors, yet to be connected with cilia, must regulate many cilia genes in these organisms. Calcium is involved in *Chlamydomonas* cilia gene regulation *via* unknown pathways (Cheshire and Keller, 1991). In addition, it will be important to identify the recently proposed repressor (Perlaza et al., 2023). Powerful molecular (Picariello et al., 2020) and omics tools now available in *Chlamydomonas* make this an excellent time to revisit previous approaches that identified mutants defective in cilia gene regulation (Lefebvre et al., 1988). Targeted disruption of candidate genes and screens for mutants unable to induce cilia genes during cilia regeneration or with constitutive expression of cilia genes in the presence of full-length cilia are likely to uncover many signaling proteins and additional transcription factors in the coming years.

## Abbreviations

IFT, intraflagellar transport; MCC, multiciliated cell; MR, mineralocorticoid receptor.
